# Application of Atomic Force Microscope to Investigate the Surface Micro-Adhesion Properties of Asphalt

**DOI:** 10.3390/ma13071736

**Published:** 2020-04-08

**Authors:** Xiaoping Ji, Jia Li, Xugang Zhai, Haiwei Zou, Bo Chen

**Affiliations:** 1 Key Laboratory for Special Area Highway Engineering of Ministry of Education, Chang’an University, Xi’an 710064, China; jixp82@163.com; 2 No.1 Municipal Administration Institute, Xianyang Planning & Design Institute, Xianyang 712000, China; 3 ShenZhen Wisdri Engineering & Research Co., Ltd., Shenzhen 518000, China

**Keywords:** surface energy, AFM, bonding coefficient, asphalt, bee-like structure

## Abstract

The surface energy and bonding coefficient of asphalt are important factors that affect the adhesion performance of asphalt/aggregate. In this study, the micro-bee-like-structure of asphalt and force curves between the microscope-probe and asphalt were measured via atomic force microscopy (AFM). To investigate the influence of asphalt properties on micro-adhesion of asphalt, five types of asphalt were used in four states: original, aged at 163 °C, immersed in water and added anti-stripping agent. The results demonstrate that the surface energy of grade 90 asphalt is greater than that of grade 70 asphalt when oil source is the same and that of modified asphalt is greater than matrix asphalt. The surface energies and bonding coefficients of asphalts decreased after aging and immersion. The surface energies of asphalts were greatly improved by adding anti-stripping agent and the bonding coefficients of the asphalts increased by 5.04–37.14% after adding an anti-stripping agent.

## 1. Introduction

With the development of high-grade highways, asphalt pavements have become the primary form of high-grade pavement in various countries. With continuous changes being made in the functional requirements of pavement, the design of asphalt pavements structure and asphalt mixture materials are becoming increasingly important [1,2]. In the design of asphalt mixture materials, the adhesion of the aggregate/asphalt interface is crucial, and has a direct impact on the durability, water resistance and strength of asphalt pavements. When the adhesion is insufficient, under the influence of load and water, the adhesion of the aggregate and asphalt interface fails, resulting in relative slip occurring between the aggregates. Finally, the adhesion force is lost and the asphalt falls away from the aggregate surface, thus affecting the durability of asphalt pavements, water damage resistance, strength and low temperature crack resistance of the asphalt mixture [3,4,5].

Since the 1930s, research on aggregate/asphalt adhesion has been a hotspot in the field of road materials with researchers around the world putting forward various testing methods and evaluation indicators. Boiling and soaking methods have been used as classical static spalling tests [6]. Subsequently, the dynamic scouring water immersion method, low temperature adhesion tests on asphalt and aggregate and other dynamic peeling tests were gradually proposed [7]. Because of the development and crossover between various disciplines, road materials science and fields such as chemistry, biology and physics are becoming increasingly closely related. Moreover, the photoelectric color pen, SHRP (Strategic Highway Research Program) static adsorption and solvent elution methods have been successively proposed [8]. However, these studies only focus on the macro testing of asphalt mixtures; therefore, it is impossible to use these methods to explain aggregate/asphalt adhesion and its failure mechanism at the micro-interface level, and these methods cannot reliably provide reference values for materials design.

Previous studies have shown that the adhesion between aggregate and asphalt is mainly caused by the micro-forces of the interface, including the physical and chemical interactions between the two phases of the interface, which are controlled by the surface microstructure and chemical composition of the material [9,10]. To quantitatively analyze the adhesion characteristics between the aggregate and asphalt on microscale, researchers worldwide have performed many studies and devised a surface energy theory. The methods for evaluating the adhesion properties of aggregate and asphalt must be simple to operate, yield reliable results, provide quantifiable indicators and be easy to compare. In 1997, Elphingstone took the lead in proposing the validity of the surface free energy theory for studying the water damage and fatigue resistance of asphalt mixtures [11]. Thereafter, Jonatha et al. studied the water damage characteristics of different polymer-modified asphalt–aggregates based on the surface energy theory [12]. Cuadr and Partal then produced modified asphalt using a castor oil prepolymer functionalized with thiourea and isocyanate, measured the surface energy parameters of the base and modified asphalt based on the surface energy theory, and further measured the adhesion performance of the asphalt and aggregate [13]. The results show that the adhesion of the modified asphalt and aggregate was higher than that of the matrix asphalt. Arabani proved that the surface energy theory can be used to measure the water stability of an asphalt mixture by analyzing the formation mechanism of the adhesion forces of asphalt mortar [14]. Alvarez prepared different asphalt mixtures using seven types of asphalt and three types of aggregates in different proportions and analyzed the adhesion effect between the asphalt and aggregate using surface energy theory [15].

Several studies on the adhesion between asphalt and aggregate based on the surface energy theory that have been carried out all over the world and the test methods used to measure the surface energy are different. Currently, the most extensively used methods are the suspension drop method [16], droplet morphology analysis [17], Du Noüy suspension ring method [16], Wilhelm slice method [16,18], inverse gas chromatography [19,20], atomic force microscopy (AFM) [21,22] and nuclear magnetic resonance (NMR) [23]. AFM is one of the newest methods that has been used for surface science testing and can be used to detect the micro-morphology, phase separation and mechanical properties (stiffness, viscosity, adhesion, and friction) of asphalt surfaces at the molecular scale. AFM has the advantages of accuracy, validity, and diversification of the test environment; therefore, it is feasible to study the asphalt surface energy of different asphalt and aggregate materials on microscale using AFM as the primary technique. Mansourkhaki studied the mechanism of the influence of a regenerant on asphalt adhesion using AFM, synthetically evaluated the physical and chemical properties of the regenerated asphalt and determined the optimum regenerant content [24]. García proposed a new micro–nano mechanical analysis method for measuring the elastic modulus and properties of asphalt based on AFM [25]. The AFM images and mechanical tests recorded using this technique show that the properties of asphalt are considerably affected by aging, oxidation and morphological changes. Liu used the mechanical properties module of AFM to select three typical observation areas in asphalt to perform AFM tests and micro-scale quantification of the mechanical properties [26]. The results show that AFM technology can be effectively used to test the modulus and adhesion properties of different asphalt and aggregate mixtures. Wang studied the influence of the chemical composition of asphalt on its micro-phase state using AFM [27]. Allen et al. determined that different chemical compositions of asphalt have different effects on the micro-properties and micro-morphology of asphalt through the AFM testing of different asphalts [28,29]. Nazzal explored the relationship between the structural characteristics of asphalt and adhesion force at the nanoscale using AFM [30].

Note that the micro-adhesion properties of aggregate/asphalt are affected by the asphalt properties, aging, moisture and modifier; moreover, all these factors are interrelated. However, in the existing literature, there has been no comprehensive rule proposed on the influence of various factors on the micro-adhesion properties of asphalt surfaces and the fact that the bee-like structure of the material is bound to have an important impact on the materials properties has not been noted. To address this gap in the literature, AFM was used in this study to measure the force curves between the microscope probe and asphalt, to measure the micro bee-like structure of the asphalt surface, to determine the surface energy parameters and to quantitatively calculate the bonding coefficients of asphalt using contact mechanics and surface energy models. The effects of the asphalt properties (such as oil source, grade and modification), aging, moisture and anti-stripping agent on the micro-adhesion properties of the surface of the material were comprehensively studied, and the influence of the micro bee-like structure on the surface energy parameters was investigated, thus providing a theoretical basis for quantification and asphalt/aggregate adhesion strength and serving as a reference for the designing materials of asphalt mixtures.

## 2. Source Material Selection

To examine the influence of the asphalt oil source, grade and modification on the surface micro-adhesion properties, five different types of asphalt were selected, namely matrix asphalts that are graded 70 and 90 and styrene–butadiene–styrene (SBS)-modified asphalt. The matrix asphalts graded 70 include SK, KL and Shell. The SBS-modified asphalt was prepared by adding 4% SBS modifier to Shell graded 70 via a high-speed shearing. The capacity of the technique was evaluated according to the Standard Test Methods of Asphalt and Asphalt Mixtures for Highway Engineering (JTJ 052-2011) of China [6]. The results are presented in Table 1.

## 3. Testing Methods and Data Evaluation

### 3.1. Testing Methods

#### 3.1.1. Asphalt Sample Preparation

**Figure 1:**
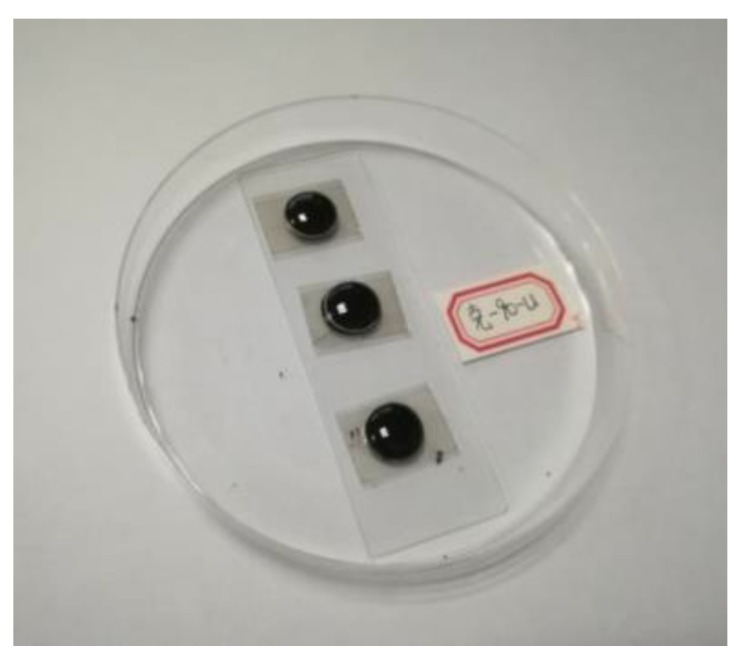
Asphalt samples.

Asphalt samples were prepared as follows. First, a rectangular mica piece 18 mm in length, 13 mm in width and 0.1 mm in thickness was placed on a glass slide, and then an iron ring with an outer diameter of 10 mm, an inner diameter of 8 mm and a height of 1 mm was placed on the mica piece. Second, the asphalt heated to 150–160 °C was dropped into the iron ring and then placed in a 150 °C oven for 20 min to make the asphalt surface smooth and flat. Then, the asphalt sample was removed from the oven together with the base and the iron ring, and the asphalt sample was placed into a plastic dish with a sealed cover to allow the sample to cool to room temperature. The sample was then placed in a sealed plastic dish before testing to prevent dust from contaminating the surface and affecting the test results. The prepared asphalt samples are illustrated in Figure 1. For each type of asphalt, three samples were prepared and tested in parallel.

#### 3.1.2. Measurement of the Microscopic Morphology and Force Curves

**Figure 2:**
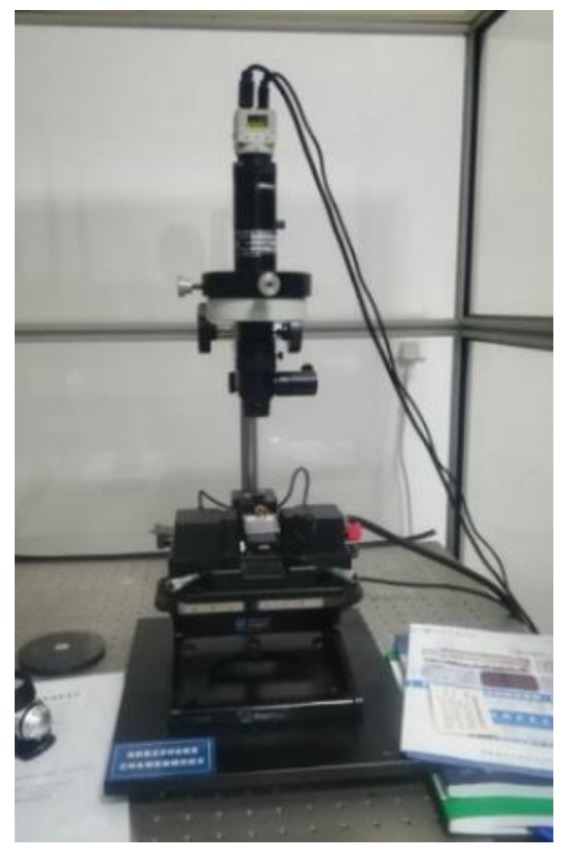
The Agilent 5400 AFM.

The Agilent 5400 AFM equipment (Agilent Technologies, Santa Clara, CA, USA) used in the experiments is shown in Figure 2. The testing process was divided into micromorphology scanning and force curve testing, the overall test steps of which are as follows. Firstly, the asphalt sample was placed on the microscope stage so that the probe was able to drop closer to the surface of the sample. Then, the scanning area and frequency were set and the sensitivity of the probe was measured. Secondly, the tapping mode was used to scan the micromorphology and measure the bee-like structure of the asphalt, as well as determine the position of the bee peaks and valleys. Finally, the force–displacement curves of the bee peaks, valleys and non-bee structure area were obtained in contact mode. The test parameters are shown in Table 2. The test probe used was a silicon probe with a radius of curvature of 7 nm, a surface energy of 1649.07 mJ/m^2^ and a Poisson’s ratio of 0.25. For details of other parameters, please refer to Table 3.

There are three types of AFM modes that can be used to scan surface micro-topography, namely the contact, peak force and tapping modes. In contact mode, the probe is directly in contact with the sample. When the hardness of the sample is considerable, the test results are relatively stable. When the hardness of the sample is less, the surface of the sample is easily damaged, resulting in test errors. In peak force mode, morphological imaging and mechanical measurements can be simultaneously performed, but the scanned sample topography lacks fidelity when the function switches. In tapping mode, the probe is in contact with the surface of the sample for a very short time, thus the resulting image has improved resolution and the probe does not damage the sample [31]. As asphalt is a soft and sticky material, the tapping mode is the best one to use to test its surface micro-topography.

**Figure 3:**
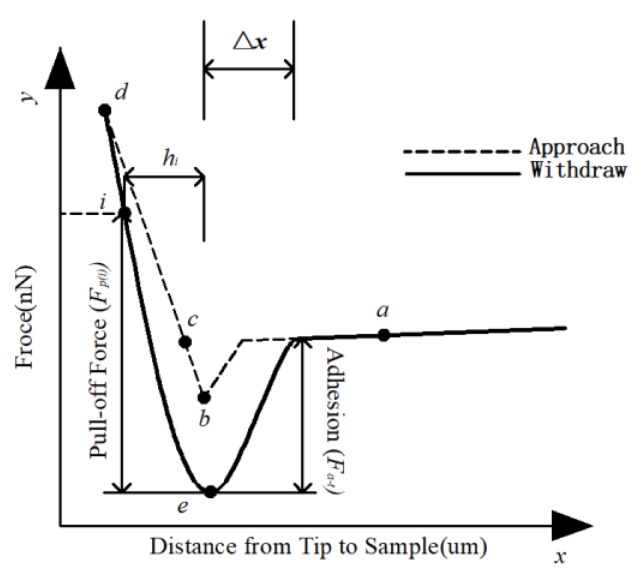
the force curve of asphalt.

The contact mode was used to measure the force curve between the probe and asphalt. There is an attraction between the atoms in the AFM probe tip and the atoms in the sample, and the attraction of various samples is different. From the force curve (such as in Figure 3), the adhesion between the sample and the probe can be measured, wherein the *a–b–c–d* path corresponds to the approach curve and the *d–e–a* path corresponds to the withdrawal curve. When the probe approaches the sample from *a*, the sample and probe are attracted together at *b*. As the probe continues to press in (*b–d*), the sample elastically deforms. After the probe reaches the set position *d*, the scanner moves in the opposite direction, and the probe begins to retreat (*d–e–a*). During the retrogression process, the probe atoms and the sample experience pull-off forces under the action of gravity, and the pull-off process ends when the probe reaches position *e*, after which the probe is kept away from the sample, but there is still adhesion between the probe and the sample until returning to its original position *a*.

In this study, the penetration depth of probe (the abscissa of point *d* of force curve) w set in AFM test. However, if the penetration depth is too large, the probe not only is attracted by the asphalt surface, but also affected by the internal adhesion of asphalt, and the force curve of asphalt surface will be disordered. Therefore, according to different positions of asphalt, the set position *d* is different, so that the probe does not press into the too deep position of asphalt and is affected by the internal adhesion of asphalt, and does not affect the calculation of the adhesion of asphalt surface, which reduces the experimental error.

### 3.2. Evaluation Indicators

#### 3.2.1. Surface Energy Parameters

Based on the AFM force curve, the surface energy of asphalt can be calculated using contact mechanics and surface energy models, the detailed steps of which are as follows:

Step 1: The adhesion force (*F_a−t_*) between the probe and asphalt was calculated using Equation (1) [32]. In the theoretical state, the displacement between the probe microcantilever and the sample is the same as the bending of the probe microcantilever exactly. However, in practice, there are elastic deformation and plastic deformation when the probe tip contacts the sample, and the probe tip press into the sample surface; thus, *θ* was quoted as the correction factor in this study, which reflects the difference between the actual bending state and the theoretical bending state of the microcantilever. *k* is the elastic constant of the probe, which is presented in Table 3, and Δx is the displacement of the probe microcantilever during the process from “non-bending state” to completely leaving the sample surface.

(1) $$F_{a-t}=\theta •k•\Delta x$$

Step 2: Classical JKR theory, which is applicable to systems with high viscosity and low rigidity, can be used to describe the interaction between the probe and the material [33]. Because asphalt is a high viscosity and low rigidity material, some scholars have applied JKR theory for research on the micro-adhesion of asphalt. Yi [21,34] and Gong [35] calculated the adhesion work using JKR contact model. According to JKR contact mechanics model, the adhesion work, *w_a−t_*, between the probe and asphalt can be calculated as shown in Equation (2):

(2) $$F_{a-t}=\frac{3}{2}\pi Rw_{a-t}$$

where *F_a−t_* is the adhesion between the probe and asphalt, *R* is the contact radius, and *w_a−t_* is the adhesion work between the probe and asphalt.

Step 3: Combining the surface energy (*γ_t_*) of the probe, the surface energy (*γ_a_*) of the asphalt was calculated using the surface energy model from the adhesion work, *w_a−t_*, as shown in Equation (3) [32,36]:

(3) $$w_{a-t}=2\sqrt{\gamma_{a}\gamma_{t}}$$

where *w_a−t_* is the adhesion work between the probe and asphalt, *γ_a_* is the surface energy of the asphalt, and *γ_t_* is the surface energy of the probe.

In the tests, nine force curves were obtained for each type of asphalt, for three regions, namely the bee peaks, valleys and other areas. The average value of the calculated surface energy was taken as the test result and the influences of the asphalt type, aging and soaking on the surface energy were analyzed. Considering the area proportion of the bee-like structure, the adhesion and surface energy values of the bee-like structure and other areas, the total adhesion and surface energy of the asphalt surface were calculated according to Equations (4) and (5).

(4) $$F_{a-t}=\sum P_{i}\times F_{a-t(i)}$$

(5) $$\gamma_{a}=\sum P_{i}\times \gamma_{a}{}_{\left(i\right)}$$

where *p_i_* represents the area ratios of the peaks, valleys and other areas, *F_a−t_*_(*i*)_ is the adhesion of the asphalt in different regions, and *γ_a_*_(*i*)_ is the energy of the asphalt in different regions.

#### 3.2.2. Bonding Coefficient

**Figure 4:**
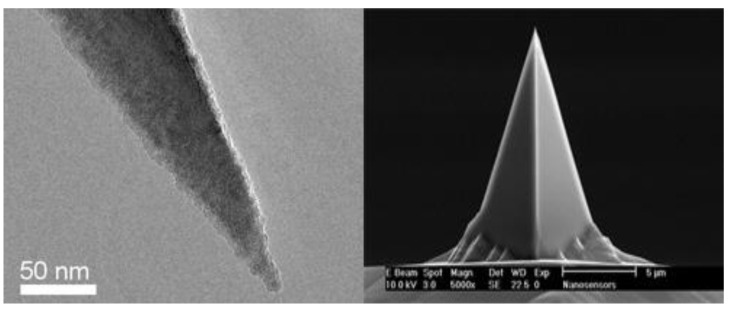
The shape of the AFM probe tip.

The withdrawal curve of the force curve (*d–e–a* in Figure 3) shows the relationship between the pull-off force and distance between the probe and asphalt during the withdrawal process. As the probe is tapered (as shown in Figure 4), the area of contact surface, *s_p_*, between the probe and the asphalt becomes smaller and its pull-off force, *F_p_*, gradually decreases when the probe is far away from the asphalt sample; however, the ratio of the two values is relatively stable. The average ratio (*k_p_*) of the pull-off force (*F_p_*) and the contact area (*s_p_*) of several points in the pull-off force curve is considered as the average bonding coefficient of the asphalt sample, which reflects the degree of adhesion between the probe and the asphalt during the pull-off process.

(6) $$k_{p(i)}=F_{P(i)}/s_{p(i)}$$

When the depth of the probe is in the range of 0.1–0.3 μm, the tip of the probe can be regarded as a cone with a cone angle of 20°, and the contact surface (*s_p_*) can be calculated using Equations (7)–(9):

(7) $$r_{p(i)}=h_{p(i)}\cdot \tan\alpha$$

(8) $$l_{p(i)}=\sqrt{r_{p(i)}{}^{2}+h_{p(i)}{}^{2}}=\frac{h_{p(i)}}{\cos\alpha }$$

(9) $$s_{p(i)}=\pi r_{p(i)}l_{p(i)}=\pi h_{p(i)}{}^{2}\frac{\tan\alpha }{\cos\alpha }$$

where *s_p_*_(*i*)_ is the contact area, *r_p_*_(*i*)_ is the contact bottom radius, *l_p_*_(*i*)_ is the contact bus length, *α* is the contact dip angle of 20°, and *h_p_*_(*i*)_ is the pressing depth.

From the AFM force curves of three regions of different asphalts, the adhesion coefficients of the different areas can be calculated. Combined with the area proportion (*P*) of the bee-like structure and other areas in the asphalt, the overall adhesion coefficient (*k*) of the asphalt surface can be calculated, as shown in Equation (10):

(10) $$k=\sum P_{i}\times k_{p}{}_{\left(i\right)}$$

where *P_i_* represents the area ratios of the peaks, valleys and other areas and *k_p_*_(*i*)_ represents the bonding coefficients of different regions of the asphalt.

## 4. Results and Discussion

### 4.1. Surface Energy and Influencing Factors

#### 4.1.1. The Influence of the Asphalt Type on the γ_a_ Value

The influences of the oil source, grade, modification and bee-like structure on the micro-adhesion properties of asphalt were analyzed. Before the test force curves were obtained, the surface micromorphology was scanned in tapping mode, and then the force curves were recorded in contact mode at a fixed point (bee-like and non-bee-like structures).

**Figure 5:**
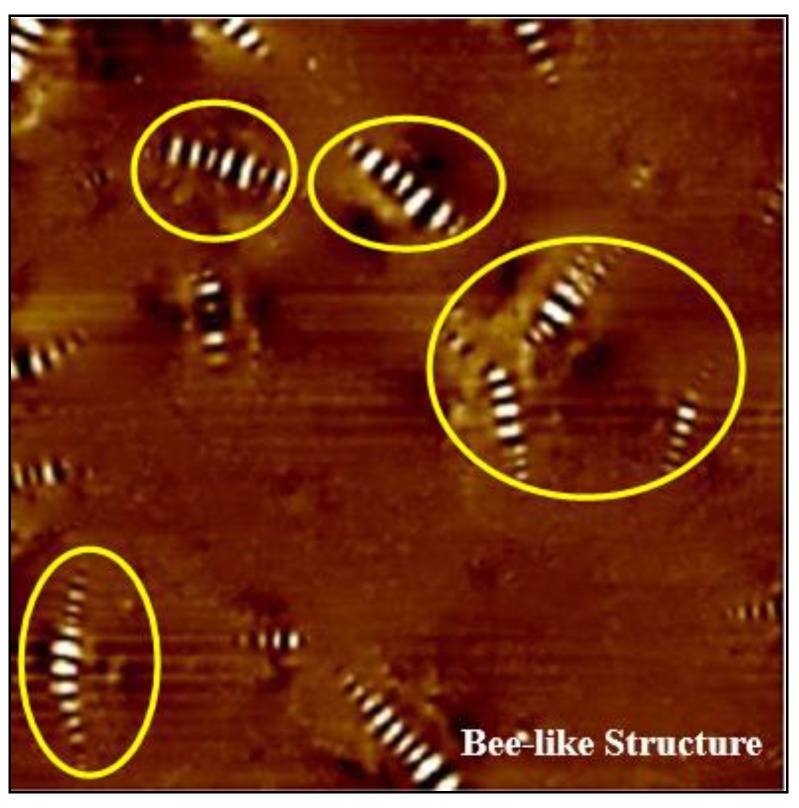
AFM Image of bee-like structures.

**Figure 6:**
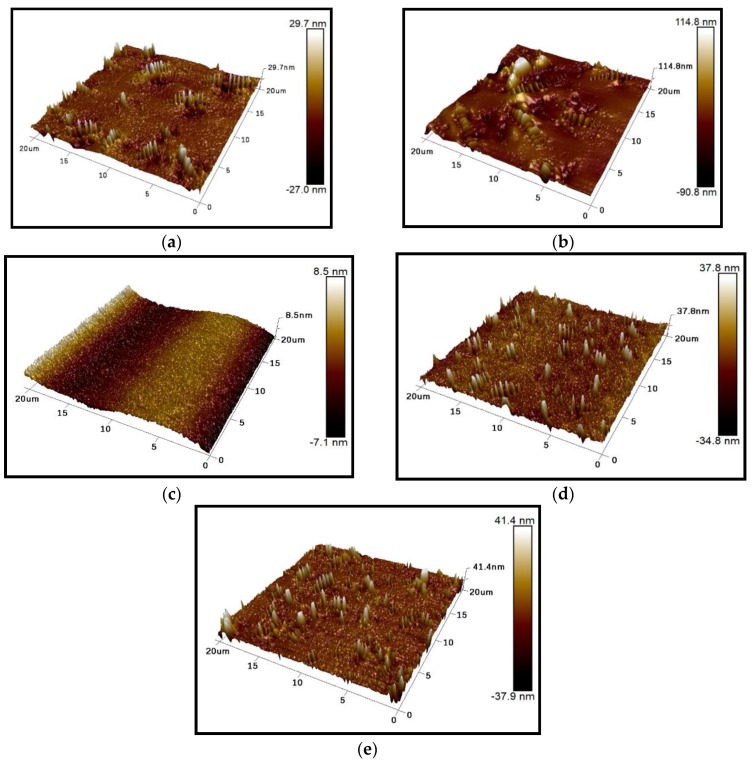
Bee-like structures of the asphalts: (**a**) SK-70; (**b**) SK-90; (**c**) KL-70; (**d**) Shell-70; and (**e**) SBS-modified.

**Figure 7:**
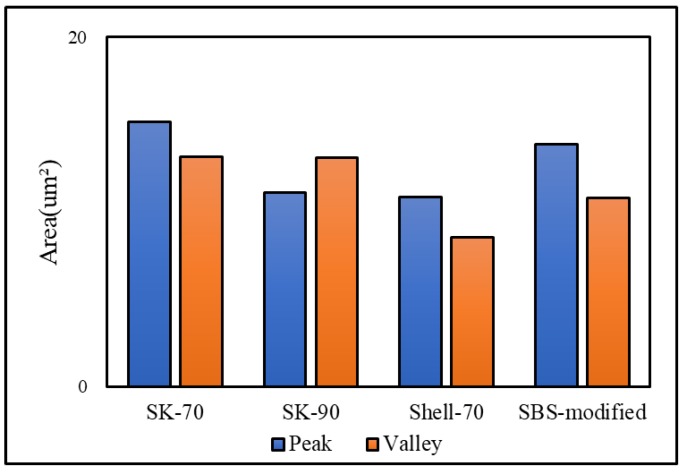
The area of bee-like structure in original asphalt.

The bee-like structure of the asphalt surface can be observed by AFM (Figure 5), and the results show that this structure affects both the micro-morphology of the material surface [37,38,39] and the micro-mechanics of the asphalt [40]. Therefore, it is necessary to investigate the effect of the bee-like structure on the surface energy. Figure 6 shows an image of the observed bee-like structure, which was imported into MATLAB to calculate and count the area of the bee-like structures in the image, the results of which are shown in Figure 7. The image shows that, while there are bee-like structures in the SK-70, SK-90, Shell-70 and SBS-modified asphalts, the number and area of bee-like structures are significantly different. KL-70 has no bee-like structures. A bee-like structure is a special feature in asphalt micro-surface.

**Figure 8:**
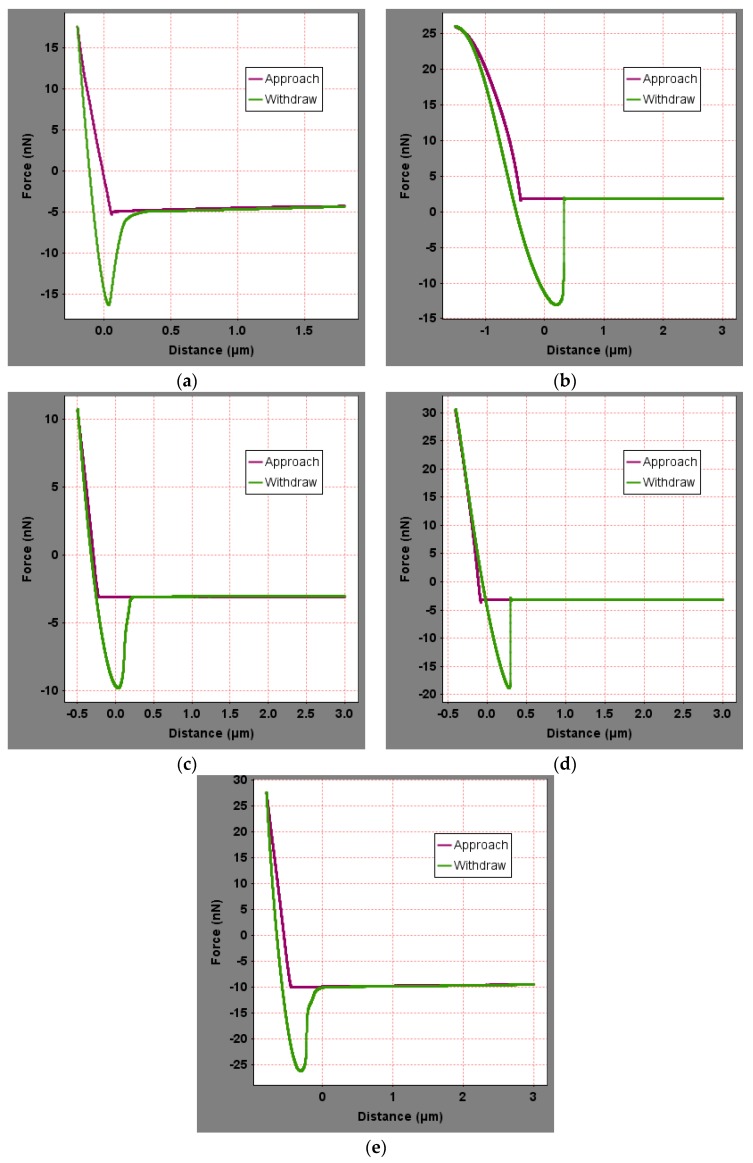
Force curves of the peak region of the bee-like structures in asphalts: (**a**) SK-70; (**b**) SK-90; (**c**) KL-70; (**d**) Shell-70; and (**e**) SBS-modified.

**Figure 9:**
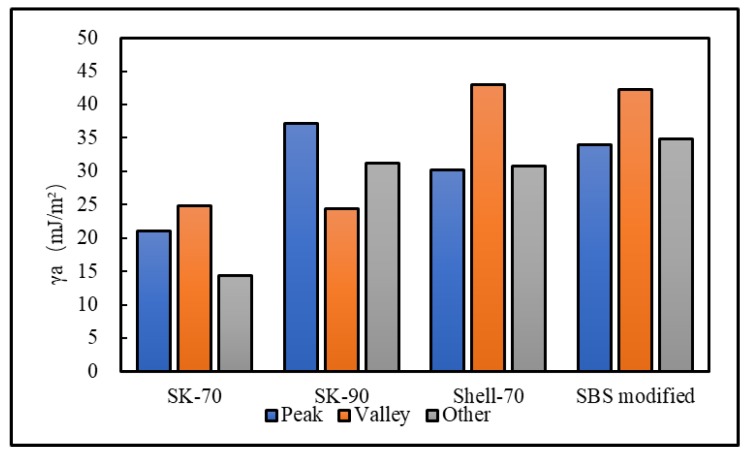
Surface energies of the different regions of the original asphalts.

**Figure 10:**
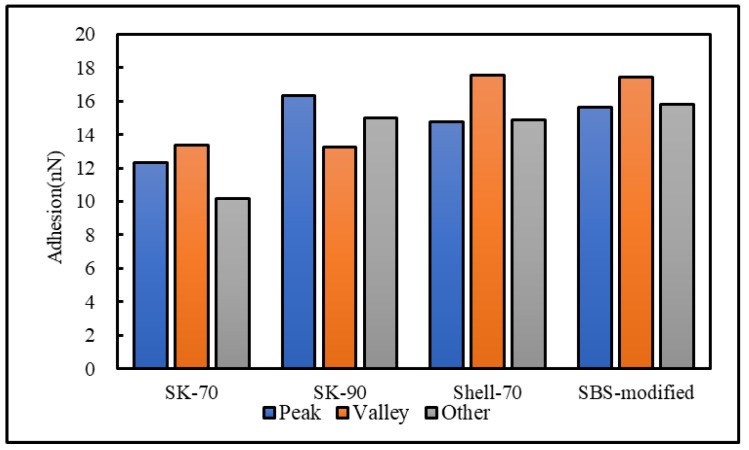
Adhesion between the probe and original asphalt in different regions.

To analyze the influence of the bee-like structure on the surface energy, AFM was used to measure the force curves of different regions of the SK-70, SK-90, KL-70, Shell-70 and SBS-modified asphalt. Figure 8 shows the force curves at the peaks of various asphalt bee-like structures. For simplicity, the force curves in the valleys and other areas are not shown in this paper. According to the force curve results, the adhesion between the asphalt and the probe *F_a−t_* can be calculated using Equation (1), and the surface energy, *γ_a_*, can be calculated using Equations (2) and (3), the results of which are shown in Figure 9 and Figure 10. The results show that the bee-valley surface energies of the SK-70, Shell-70 and SBS-modified asphalt are greater than those of the bee-peaks and other areas; however, the surface energy of the SK-90 asphalt bee-valley is less than that of the bee-peak and other areas. However, because of limitations in science and technology, there is no method that can be used to analyze the chemical composition and mechanism of the formation of the different regions of the bee-like structure; therefore, it is difficult to explain the singularity of the surface energy in different regions.

The total adhesion and surface energies of the different asphalt surfaces were calculated by combining the surface energy values and ratios of the bee-like structures to other areas, the results of which are shown in Table 4.

The results demonstrate that the surface energy of SK-90 asphalt is higher than that of SK-70 asphalt when the oil source is the same and the grade is different. When the grade is the same and the oil source is different, the surface energy ordering is Shell-70 > SK-70 > KL-70, i.e., the surface energy of modified asphalt is higher than that of matrix asphalt, and the surface energy of KL-70 asphalt without a bee-like structure is the lowest. According to the analysis of the five types of asphalt, as shown in Figure 6, the microstructures of asphalt are quite different. The special morphology of the bee structure on the asphalt surface inevitably has an impact on the asphalt micro-adhesion properties, therefore explaining why the surface energy of KL-70 without a bee-like structure is the lowest. The area, quantity and dispersion of the bee-like structures in the SBS-modified materials are greater than those in the other asphalt; thus, their surface energies are higher. The surface energy of the SBS-modified is the greatest because this type of asphalt is primarily composed of non-polar hydrocarbons. After SBS is added, the light oil components are absorbed by SBS, with some of them transforming into gum and asphaltene materials, which have strong polarity and high viscosity [41]. Therefore, the polar components of SBS-modified asphalt increase and the adhesion work produced by the polar components is greater, which effectively improves the adhesion between the asphalt and aggregate.

#### 4.1.2. The Influence of Aging on the γ_a_ Value

**Figure 11:**
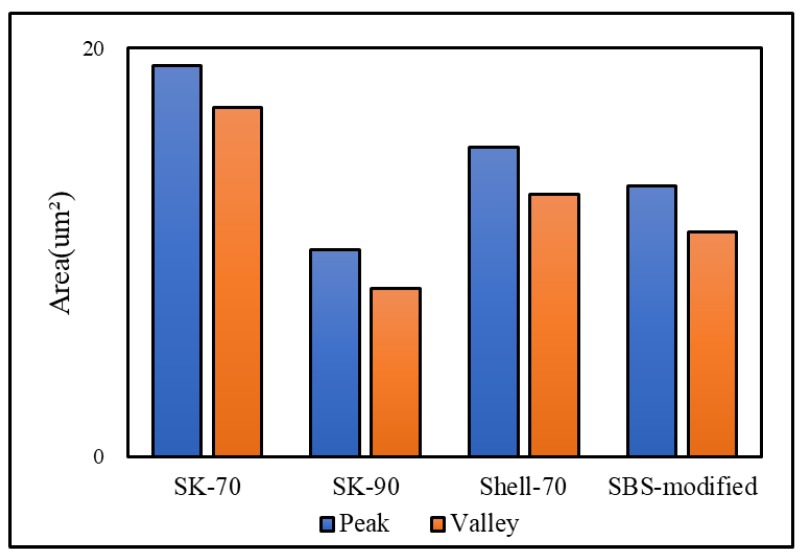
The area of bee-like structure in aged asphalt.

Previous studies show that the aging process of asphalt is often accompanied by the volatilization of light components and some slight chemical changes [42]; therefore, the number of bee-like structures and the area they occupy changes, and the statistical area is shown in Figure 11.

**Figure 12:**
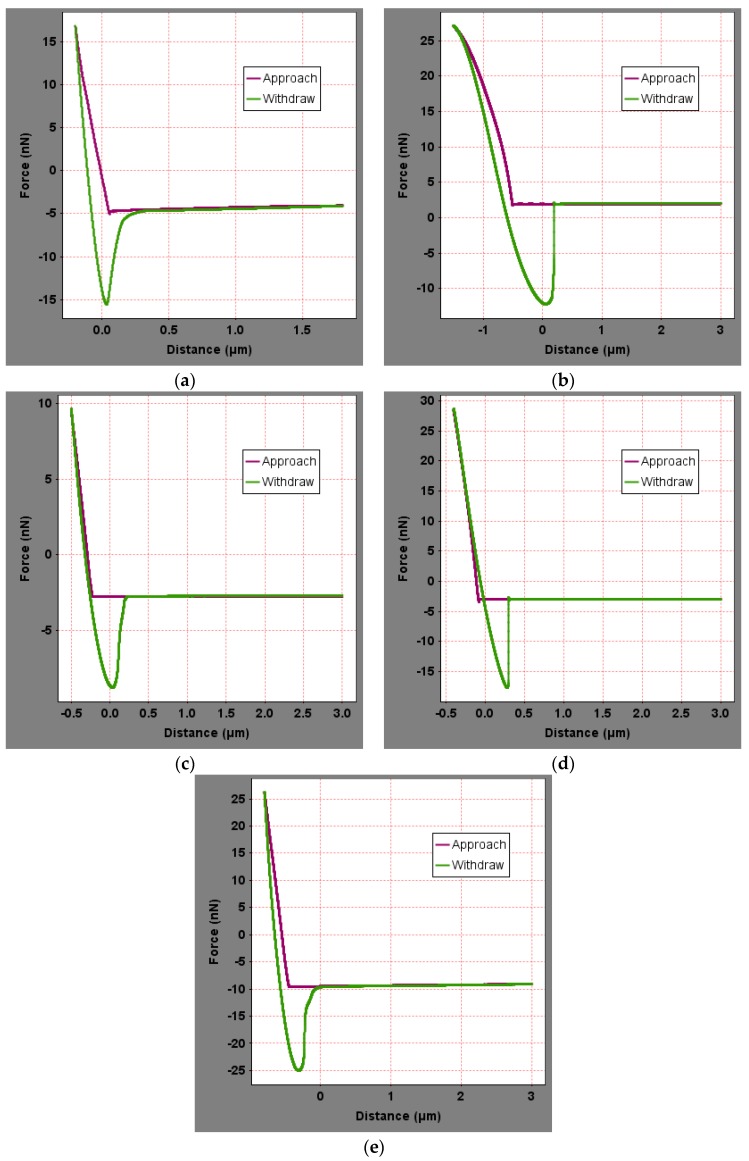
Force curves of the valley region of the bee-like structures in aged asphalts: (**a**) SK-70; (**b**) SK-90; (**c**) KL-70; (**d**) Shell-70; and (**e**) SBS-modified.

**Figure 13:**
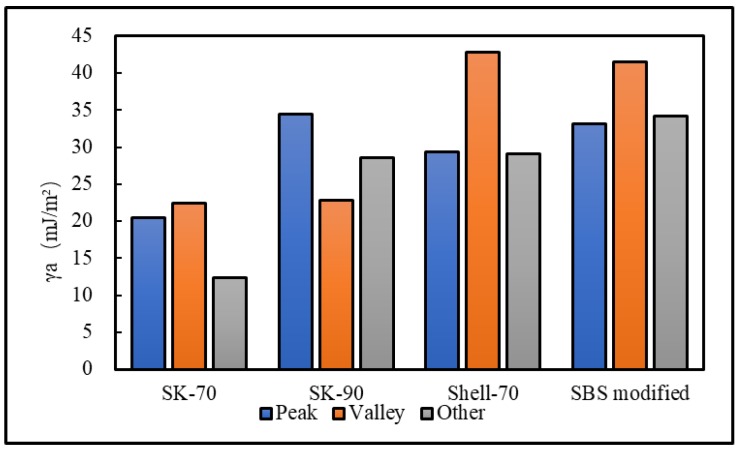
Surface energies of different regions in the aged asphalts.

**Figure 14:**
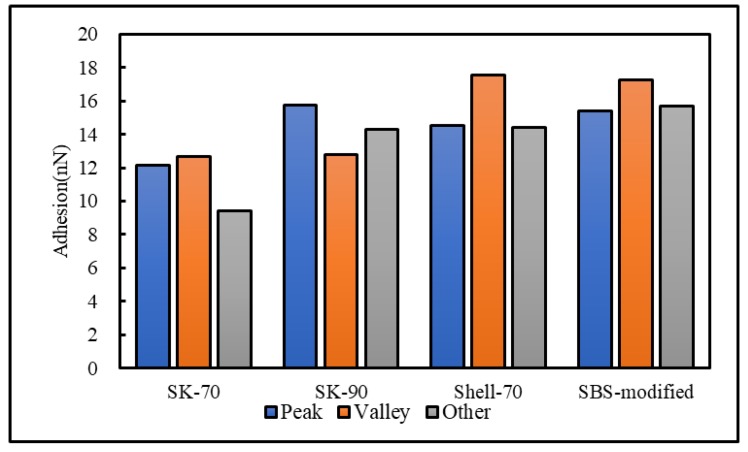
Adhesion between the probe and the aged asphalt in different regions.

To explore the influence of aging on the surface energies of the asphalt, AFM was used to measure the force curves of the five types of aged asphalt studied in this work. Figure 12 shows the force curves of the bee valley regions in the different asphalt. For simplicity, the force curves of the peaks and other areas of the asphalt are not shown in this paper. The results of the calculation of the adhesion and surface energies of the different areas of the asphalt studied in this study are shown in Figure 13 and Figure 14. Compared with the results in Figure 9 and Figure 13, the surface energies of the peaks for the bee-like structures of the four asphalt decreased by 3.06%, 7.33%, 3.07% and 2.54%, and the surface energies of the bee valleys decreased by 9.65%, 6.53%, 0.29% and 2.05% compared to the surface energies of the other areas, which decreased by 14.02%, 8.75%, 5.62% and 1.51%. The surface energies of other areas of the matrix asphalts demonstrated a considerable decrease than those of the bee peaks and bee valleys, and the surface energies of the modified asphalt bee peaks decreased more than those of the bee valleys and other areas.

The asphalt surface energies before and after aging were compared, the results of which are shown in Table 5. The results demonstrate that the surface energies of the SK-70, SK-90, KL-70, Shell-70 and SBS-modified asphalt decreased by 12.00%, 8.47%, 7.62%, 4.91% and 1.55%, respectively. The surface energy of SBS-modified asphalt showed the lowest decrease, while the surface energy of KL-70 asphalt showed the highest decrease. Previous studies have shown that asphaltene is the most polar substance in asphalt and that its existence is closely related to the surface energy of asphalt [43,44]. In the aging process of asphalt, the asphaltene content increases. To maintain a stable asphalt system, asphaltene molecules aggregate together through charge transfer or hydrogen bonding to further reduce the energy of the system. This results in a decrease in the degree of dispersion of the asphaltene in the asphalt, thus reducing the polarity of the asphalt and further reducing the surface energy, which are the same conclusions reached by Li [45] and Jada [46].

#### 4.1.3. The Influence of Water on the γ_a_ Value

**Figure 15:**
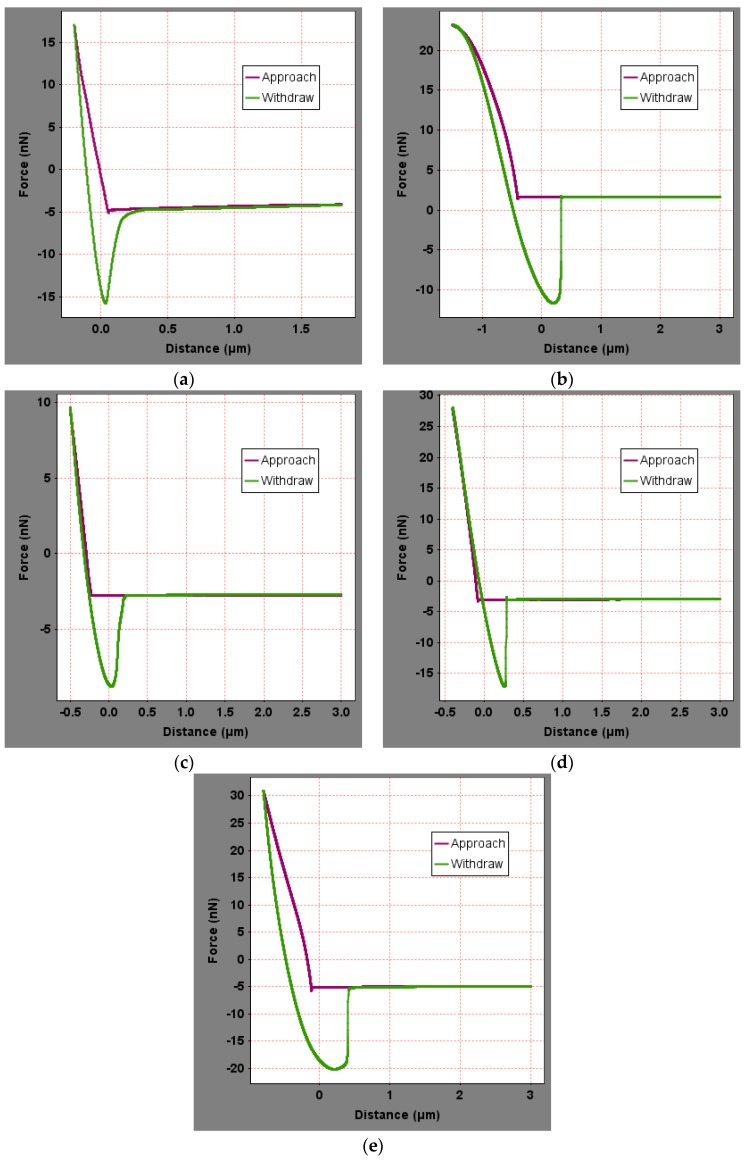
Force curves of the other regions of the bee-like structures in soaked asphalts: (**a**) SK-70; (**b**) SK-90; (**c**) KL-70; (**d**) Shell-70; and (**e**) SBS-modified.

**Figure 16:**
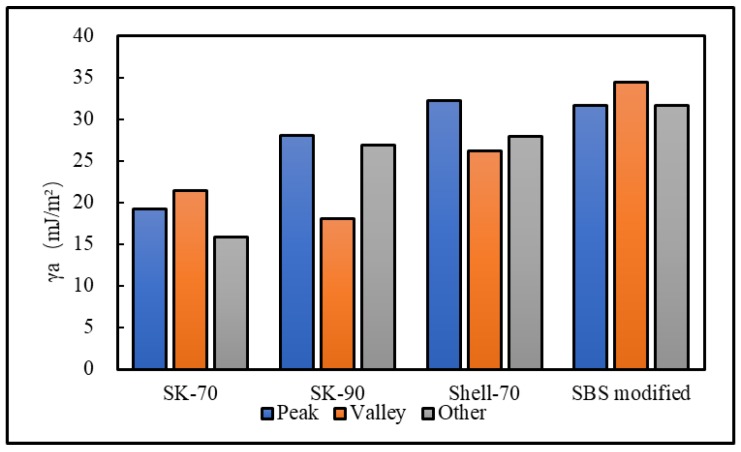
Surface energies of different regions of the soaked asphalts.

**Figure 17:**
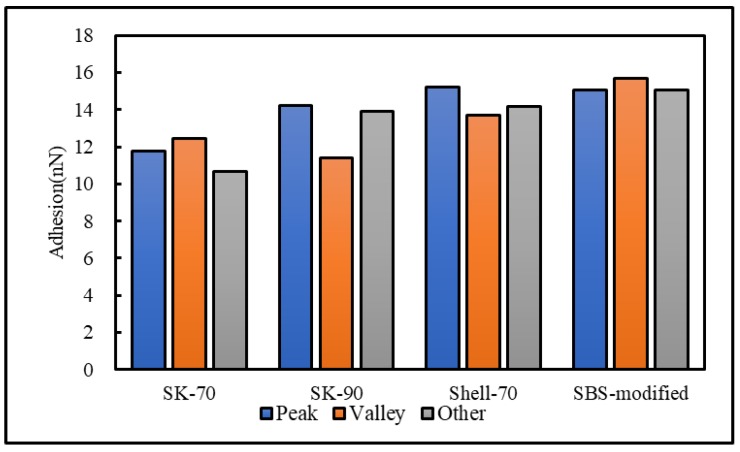
Adhesion between the probe and soaked asphalts in different regions.

Generally, there is no obvious difference in the surface micro-morphology before and after asphalt immersion [47]. Therefore, there are no changes in the areas and quantities of the bee-like structures. The statistical areas are shown in Figure 7. AFM was used to measure the force curves of the SK-70, SK-90, KL-70, Shell-70 and SBS-modified asphalts after soaking them for 24 h in water. Figure 15 shows the force curves of the five types of asphalt in the other regions of the bee-like structure. For simplicity, the force curves of the peak and valley areas are not shown in this work. The results of the calculation of the adhesion and surface energies of the asphalts in different regions are shown in Figure 16 and Figure 17.

According to Figure 16 and Figure 17, the decreased range of surface energy in bee valley areas is larger than that in other areas, which are 13.28%, 24.40%, 38.89% and 18.72%. From the change in the surface energy of the bee valley, the contact area with water will have an impact on the change in the surface energy. Furthermore, according to the change in the surface energy of the bee peak, the contact area with water is not the only factor that affects the surface energy. The results of the calculation of the overall surface energies of the asphalts after immersion are shown in Table 6. The surface energies of SK-90, Shell-70 and SBS-modified asphalts decreased by 14.66%, 9.57% and 8.87%, respectively. Moreover, the presence of water on the asphalt surface reduces the surface energy of asphalt because there are nonpolar (lipophilic) and polar groups (hydrophilic) on the surface of the asphalt. Note that water molecules and polar groups gather to form micro activation centers. The negative oxygen and nitrogen atoms and water molecules in asphalt form hydrogen bonds, and the surface energy of the asphalt decreases [48,49]. On the contrary, the surface energy of SK-70 and KL-70 increased by 7.60% and 25.30%, respectively, which may be due to the fact that 24-h immersion did not make the water completely penetrate into the asphalt and soften the asphalt, resulting in abnormal surface energy test results [50].

#### 4.1.4. The Influence of an Anti-Stripping Agent on the γ_a_ Value

**Figure 18:**
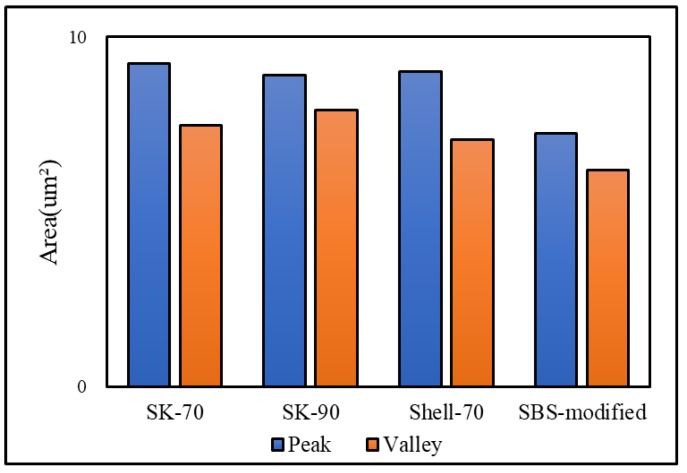
The area of bee-like structure in asphalt doped with RAA.

The microstructure of an asphalt surface changes after adding 0.5% Willis RAA asphalt anti-stripping agent and the statistical areas of the bee-like structure are shown in Figure 18.

**Figure 19:**
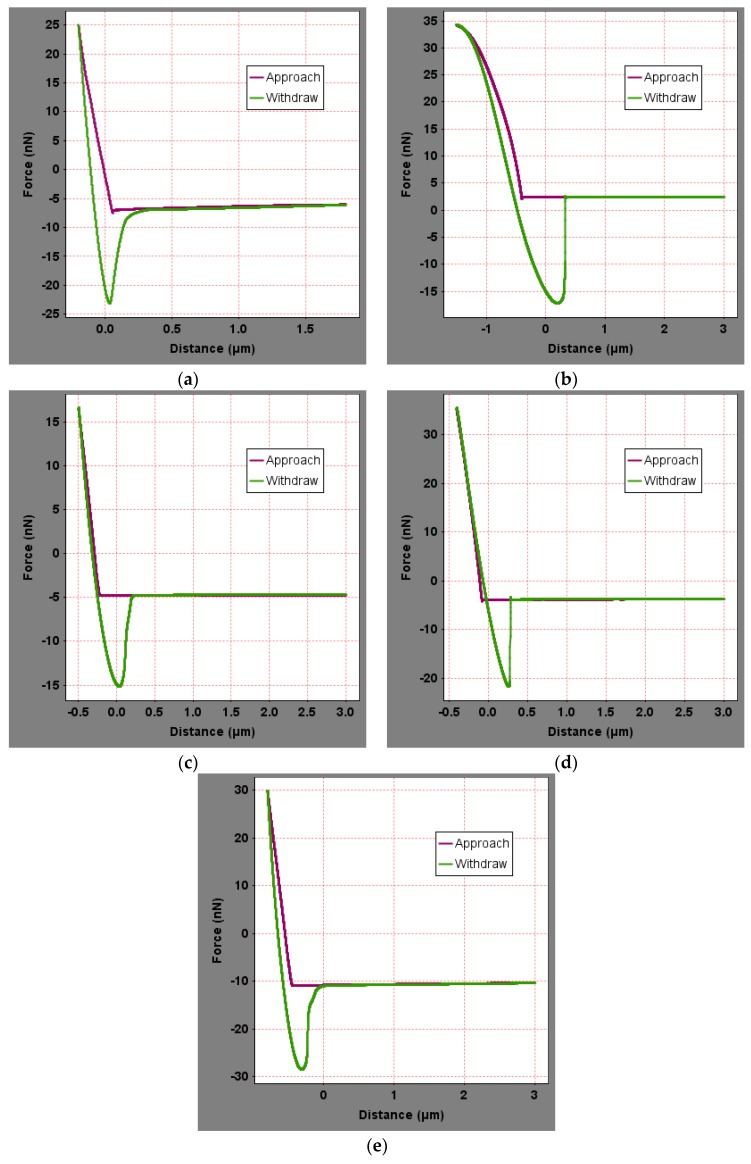
Force curves of the peak region of the bee-like structures in asphalts doped with RAA: (**a**) SK-70; (**b**) SK-90; (**c**) KL-70; (**d**) Shell-70; and (**e**) SBS-modified.

**Figure 20:**
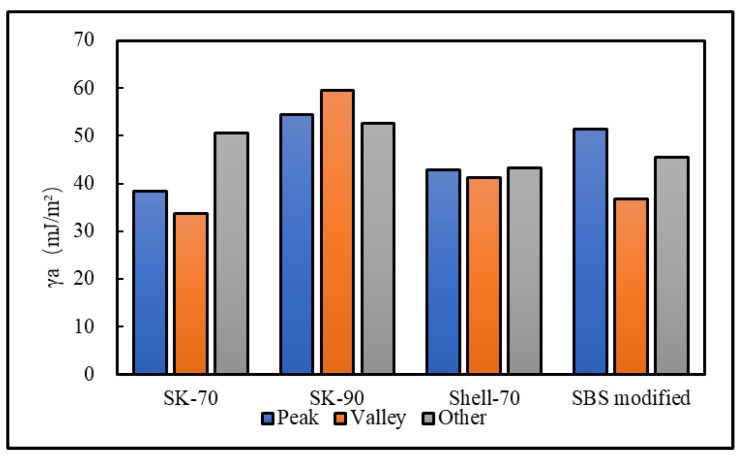
Surface energies of different regions of the different asphalts doped with RAA.

**Figure 21:**
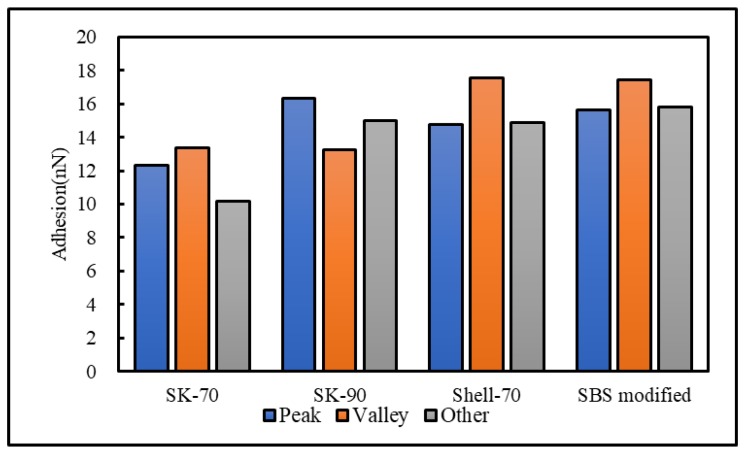
Adhesion between the probe and the different asphalts doped with RAA in the different regions.

AFM was used to measure the force curves of the SK-70, SK-90, KL-70, Shell-70 and SBS-modified asphalts after adding an anti-stripping agent. Figure 19 shows the force curves of the various asphalts at the peak of the bee-like structure. For simplicity, the force curves of the valleys and other areas are not shown in this study. The surface energies of different areas were calculated, as shown in Figure 20 and Figure 21. The results show that the surfaces of the bee peaks, bee valleys and other areas tend to be the same after adding an anti-stripping agent because the anti-stripping agent is evenly distributed on the asphalt surface such that the surface energies of the different areas of the asphalt tend to be the same.

The results of the overall surface energies of the asphalts are shown in Table 7.

Table 7 shows that the surface energies of the different types of asphalts increase by 272.74%, 79.74%, 123.29%, 42.36% and 34.15% after adding the RAA anti-stripping agent. RRA anti-stripping agent is the mixture of alkyl and alkaline amines. This indicates that the agent obviously improves asphalts with a low surface energy because asphalt, a high molecular hydrocarbon, exhibits little polar activity, and its surface energy is consisted of polar energy and non-polar energy. After adding the anti-stripping agent, the non-polar surface energy of the asphalt binder occurs a brief increase, thus improving its total surface free energy [50].

### 4.2. Bonding Coefficient

According to the withdrawal curves within the range of the depression depth, the pull-off force, *F_p_*, values were read at different times, the depression depth, *h_i_*, was read at the corresponding time, and the contact surface, *s_i_*, was calculated according to Equations (5)–(7). The bonding coefficient, *k_p_*, of different areas was then calculated using Equation (8), and then the adhesion coefficient, *k*, of asphalt was calculated according to the area proportions of the different regions, as shown in Table 8.

Table 8 shows that the bonding coefficients of the SK-70, SK-90, KL-70, Shell-70 and SBS-modified asphalts are 1.34, 0.70, 1.29, 1.25 and 2.38 MPa, respectively. In terms of grade, the bonding coefficient of grade 70 asphalt is greater than that of grade 90 asphalt, whereas that of SBS-modified asphalt is much greater than that of matrix asphalt. Note that the bonding coefficients of asphalts of the same grade and different oil sources are similar, whereas those of SK-70, SK-90, KL-70, Shell-70 and SBS-modified asphalts after aged for 85 min are 1.26, 0.64, 1.22, 1.19 and 2.31, respectively. Compared with the original asphalt, the bonding coefficient of SK-90 is reduced by 8.57%, a considerable decrease amongst the different materials; however, that of the SBS-modified asphalt is reduced by 2.94%, representing the smallest decrease. The range of decrease of the three types of grade 70 asphalt is between 4.80% and 5.97% because the polarity of the asphalt system gradually decreases during the aging process; therefore, the adhesion between the asphalt and the probe also decreases.

The bonding coefficients of the SK-70, SK-90, KL-70, Shell-70 and SBS-modified asphalt immersed in water are 1.21, 0.62, 1.17, 1.14 and 2.33 MPa, respectively. Compared with the original asphalt, the bonding coefficient of the SK-90 asphalt is reduced by 11.43%, representing the largest decrease, and the bonding coefficient of the SBS-modified asphalt is reduced by 2.10%, representing the smallest decrease. These abovementioned results show that SBS-modified asphalt has good water stability. Generally, the surface energy of asphalt is expected to reduce after soaking in water, and then the adsorption capacity is weakened.

After adding the anti-stripping agent, the bonding coefficients of the SK-70, SK-90, KL-70, Shell-70 and SBS-modified asphalt are 1.53, 0.96, 1.51, 1.43 and 2.50 MPa, respectively. Compared with original asphalt, the bonding coefficient of the SK-90 asphalt increased by 37.14%, representing a considerable increase, whereas the bonding coefficient of the SBS-modified asphalt increased by 5.04%, representing the smallest increase because the anti-stripping agent is an active substance. Therefore, the addition of the anti-stripping agent improved the polarity of the asphalt surface, thus enhancing the adhesion of the asphalt surface.

## 5. Conclusions

(1) AFM testing technology can be used to test the adhesion of asphalt surface at micro-scale, and then calculate its surface energy. The force curve measured by AFM can reflect the micro adsorption relationship between the tip of probe and asphalts.
(2) When the oil source is the same and the grade is different, the surface energy of the grade 90 asphalt is greater than that of grade 70 asphalt, whereas, when the grade is the same and the oil source is different, the surface energy of KL-70 asphalt without a bee-like structure is the least and the surface energy of modified asphalt is greater than that of matrix asphalt. Because of the different chemical compositions of the bee-like structures, the surface energies are different. The larger is the grade, the smaller is the bonding coefficient of the asphalt, with the bonding coefficient of the modified asphalt being greater than that of matrix asphalt.
(3) After aging, the surface energies of the asphalts decreased by 1.55–12.00%, the surface energies of the other areas of the matrix asphalt decreased more than those of the bee peaks and valleys, the surface energies of the modified asphalt bee peaks decreased more than those of the bee valley and other areas, and the bonding coefficients of the asphalts decreased by 4.80–5.97%.
(4) After soaking, the surface energies of the asphalts decreased by 8.87–14.66%, and the surface energies of the bee valley region decreased to the largest extent, with the bonding coefficients of the asphalts decreasing by 2.10–11.43%.
(5) After adding an RAA anti-stripping agent, the surface energies of the asphalts increased. The surface energy growth rate of the matrix asphalt is higher than that of the modified asphalt, and the surface energies of the different areas tend to be the same, where the bonding coefficients of the asphalts increased by 5.04–37.14%.

## Figures and Tables

**Table 1:** Performance Index of Asphalt.

Asphalt	25 °C Penetration (0.1 mm)	Ductility (cm)	Softening Point (°C)	After RTFOT (163 °C, 85 min)			60 °C Dynamic Viscosity (Pa·s)
				Penetration Ratio (%)	Ductility (cm)	Mass Loss (%)	
SK-70	79	76.9	50.0	71.3	7.4	−0.27	253.52
SK-90	93	110.2	47.5	60.4	8.7	−0.78	163.72
KL-70	68	84.4	46.0	67.3	7.4	−0.30	503.72
Shell-70	77	42.6	50.0	62.4	7.3	−0.49	203.45
SBS-modified	71	78.4	77.0	83.7	26.2	−0.47	10328.21

**Note:** The ductility testing temperatures used for the unmodified and SBS-modified asphalts were 10 and 5 °C, respectively. RTFOT, Rotate Thin-Film Oven Test.

**Table 2:** AFM scanning parameters.

Set Point (V)	Sample/Line	Measurement Area (μm^2^)	Scanning Frequency (Hz)	Feedback Gain (I)	Feedback Gain (P)
−0.12–5	256	20 × 20	0.9	0.5	0.8

**Table 3:** Parameters of the AFM probe.

Probe Type	Material	Depth (μm)	Radius (nm)	Length (μm)	Width (μm)	Frequency (kHz)	K (N/m)
PPP-NCST-20	Si_3_N_4_	2.8	7	150	27	160	7.4

**Table 4:** The overall surface energies and adhesion forces between the probe and asphalts.

Index	SK-70	SK-90	KL-70	Shell-70	SBS-Modified
*F_a−t_* (nN)	10.36	14.96	6.85	14.92	15.84
*γ_a_* (mJ/m^2^)	15.00	31.21	6.53	31.03	34.96

**Table 5:** The overall surface energy of different asphalts before and after aging.

Index	Condition	SK-70	SK-90	KL-70	Shell-70	SBS-Modified
*F_a−t_* (nN)	Original	10.36	14.96	6.85	14.92	15.84
	Aged for 85 min	9.70	14.32	6.58	14.54	15.71
*γ_a_* (mJ/m^2^)	Original	15.00	31.21	6.53	31.03	34.96
	Aged for 85 min	13.20	28.56	6.03	29.50	34.42

**Table 6:** The overall surface energies of the different asphalts before and after immersion in water.

Index	Condition	SK-70	SK-90	KL-70	Shell-70	SBS-Modified
*F_a−t_* (nN)	Original	10.36	14.96	6.85	14.92	15.84
	Immersed in water	10.76	13.82	7.67	14.19	15.10
*γ_a_* (mJ/m^2^)	Original	15.00	31.21	6.53	31.03	34.96
	Immersed in water	16.15	26.61	8.19	28.06	31.77

**Table 7:** the overall surface energies of the original asphalts and those doped with RAA.

Index	Condition	SK-70	SK-90	KL-70	Shell-70	SBS-Modified
*F_a−t_* (nN)	Original	10.36	14.96	6.85	14.92	15.84
	Doped with RAA	18.94	19.45	10.23	17.61	18.06
*γ_a_* (mJ/m^2^)	Original	15.00	31.21	6.53	31.03	34.96
	Doped with RAA	55.93	56.09	14.58	44.17	46.89

**Table 8:** Bonding coefficients of the different asphalts’ bonding coefficients (MPa).

Asphalt	SK-70	SK-90	KL-70	Shell-70	SBS-Modified
Original	1.34	0.70	1.29	1.25	2.38
Aged for 85 min	1.26	0.64	1.22	1.19	2.31
Immersed in water	1.21	0.62	1.17	1.14	2.33
Doped with anti-stripping agent	1.53	0.96	1.51	1.43	2.50
